# Field Evaluation of Malaria Microscopy, Rapid Malaria Tests and Loop-Mediated Isothermal Amplification in a Rural Hospital in South Western Ethiopia

**DOI:** 10.1371/journal.pone.0142842

**Published:** 2015-11-10

**Authors:** Juan Cuadros, Ramón Pérez-Tanoira, Laura Prieto-Pérez, Ines Martin-Martin, Pedro Berzosa, Vicenta González, Gebre Tisiano, Seble Balcha, José Manuel Ramos, Miguel Górgolas

**Affiliations:** 1 Department of Microbiology, Universitary Hospital Príncipe de Asturias, Alcalá de Henares, Madrid, Spain; 2 Department of Medicine and Laboratory, Gambo Rural General Hospital, Kore, West-Arsi, Gambo, Ethiopia; 3 Division of Infectious Diseases, Fundación Jiménez Díaz, Madrid, Spain; 4 Department of Medicine, Universidad Autónoma de Madrid, Madrid, Spain; 5 Department of Otorhinolaryngology-Head and Neck Surgery, University of Helsinki and Helsinki University Hospital, Helsinki, Finland; 6 Medical Entomology Unit, Department of Parasitology, National Centre of Microbiology, Health Institute Carlos III, Majadahonda, Madrid, Spain; 7 National Centre of Tropical Medicine, Health Insitute Carlos III, Madrid, Spain; 8 Department of Internal Medicine, Hospital General Universitario of Alicante.Miguel Hernández University, Elche, Alicante, Spain; Centro de Pesquisa Rene Rachou/Fundação Oswaldo Cruz (Fiocruz-Minas), BRAZIL

## Abstract

**Background:**

In up to one third of the hospitals in some rural areas of Africa, laboratory services in malaria diagnosis are limited to microscopy by thin film, as no capability to perform thick film exists (gold standard in terms of sensitivity for malaria diagnosis). A new rapid molecular malaria diagnostic test called Loop-mediated isothermal DNA amplification (LAMP) has been recently validated in clinical trials showing exceptional sensitivity and specificity features. It could be a reliable diagnostic tool to be implemented without special equipment or training.

**Objective:**

The objective of this proof of concept study was to confirm the feasibility of using LAMP technique for diagnosis of malaria in a rural Ethiopian hospital with limited resources.

**Methodology/Principal Findings:**

This study was carried out in Gambo General Hospital, West Arsi Province (Ethiopia), from November 1^st^ to December 31^st^ 2013. A total of 162 patients with a non-focal febrile syndrome were investigated. The diagnostic capability (sensitivity, specificity, positive predictive and negative predictive values) of rapid malaria tests and microscopy by thin film was evaluated in comparison with LAMP. Eleven (6.79%) out of the 162 patients with fever and suspected malaria, tested positive for LAMP, 3 (1.85%) for rapid malaria tests and none of the eleven cases was detected by thin film microscopy.

**Conclusions/Significance:**

LAMP can be performed in basic rural laboratories without the need for specialized infrastructure and it may set a reliable tool for malaria control to detect a low level parasitemia.

## Introduction

In many areas of Ethiopia, malaria laboratory diagnosis is still based on microscopy. Limitations of blood smear microscopy contributed to failure of the 1950–1960s WHO Global Programme to Eliminate Malaria [1]. Recent studies have shown that many clinical laboratories use only thin films [2] with low sensitivity and virtually no quality control on the results [3], which could affect both to sensitivity and specificity of the diagnostic test. These results could be even poorer when only thin films are prepared in recycled slides in laboratories without quality control and no periodical training for lab technicians[4]. This situation could be similar among most of the health centres of low resource African countries where no reliable rapid malaria tests (RDTs) are available [5]. RDTs are provided in Ethiopia for free to the health posts where microscopy is not feasible, after field studies showed the reliability of some kits for detecting both *Plasmodium falciparum* and *Plasmodium vivax* [2]. Therefore, some hospitals equipped with microscopes are not using RDT kits, so malaria diagnosis relies only on microscopy which could yield a low standard diagnosis in these rural settings.

Gambo Hospital is located at 2.200 m altitude above sea level in the Oromia region, a traditionally malaria-free area. However, many patients affected with malaria coming from the lower slopes of the Rift Valley, at lower altitudes, are assisted and treated in this Hospital. Malaria incidence is lower during the dry season, and higher right after the rainy season when mosquitoes spring up. In this area, a period of low incidence during the previous seasons might have aggravated the events, possibly due to a low level of immunity in the affected population [6].

Studies based in conventional PCR performed in external laboratories are suitable for occasional evaluations, but are uneconomical and require shipping of specimens to reference laboratories. Results are appropriate for evaluating new technologies, but PCR is not an useful tool for internal quality control programs. However, a new rapid and simple molecular diagnostic test called Loop-mediated isothermal DNA amplification (LAMP), does not require special equipment or space distribution in the laboratories, and provide results within 60 minutes [7–8].

A field study was designed in order to compare the standard diagnostic microscopy performed in Gambo Hospital (thin blood film), RDT and LAMP [9] on site. The objective of this study was to assure the sensitivity and specificity of standard microscopy and RDTs in the field as well as checking the feasibility of performing a simple molecular method with no special equipment requirements.

## Material and Methods

### Study Population and Selection Criteria

The study population included every patient referred to the hospital laboratory for malaria diagnosis who was older than two years and attended to the outpatient Department of Gambo Hospital with suspected uncomplicated malaria (axillary temperature > 37.5°C or report of fever in the previous 48 hours) and who did not meet any of the exclusion criteria.

Exclusion criteria were: fever ≥ 3 weeks, antimalarial treatment in the previous week, apparent focal infection (e.g., pharyngitis, meningitis, or urinary tract infection), infection that could be definitively diagnosed clinically (e.g., mumps, croup, varicella, parvovirus, measles, or rubella) and patients who had received an immunization in the preceding 48 hours or blood products in the previous 6 months.

The study was performed between November 1st and December 31st 2013 for detection of malaria infection in febrile symptomatic individuals during the dry season, in a time with a estimated higher incidence of malaria in this area.

Ethics committee approvals were obtained from both the local Research and Publication Committee of the Gambo General Hospital and the Health Unit and Ethical Review Committee of the Ethiopian Catholic Secretary (GH/MSMHF/706). We ensured that the study protocol conformed to the ethical guidelines of the 1975 Declaration of Helsinki as reflected in the approval by the institution’s human research review committee. We also made sure that oral informed consent was obtained from each patient to participate on a voluntary basis in the study and a questionnaire with complete epidemiological and clinical questions was filled out. Oral informed consent was recorded in each questionnaire and was obtained from the next of kin, caretakers, or guardians on behalf of the minors/children enrolled in this study. Written consent no was obtained because there is a high rate of illiteracy in the population. No economic compensation was granted for participating in the study. All the data were treated confidentially and anonymized.

### Sample Procedure

Whole blood was collected from each patient at the time of enrolment by digital pricking; finger prick was performed in the fingerpad of the third or fourth fingers, previously cleaned with 70% ethylic alcohol. This sample was used to carry out routine blood smears, RDTs and LAMP test. Routine blood smears were read by the health facility laboratory personnel as usual; both microscopy and RDT results were provided to the health facility physicians so they could be used for management of the patient according to the usual standard of care. Due to the low sensitivity of thin film, RDT results were given priority over microscopy.

LAMP results were obtained for the purpose of the study exclusively, and were not used for clinical management. RDTs used were those recommended for an endemic area as Ethiopia, where *P*. *falciparum* and *P*. *vivax* are co-endemic (VIKIA^®^ Malaria Ag Pf/Pan) and contained the histidine rich protein-2 (HRP-2) and pan-malarial lactate dehydrogenase (LDH).

The standard operational procedures of the Loopamp^®^ MALARIA Pan/Pf Detection Kit (FIND, Switzerland, Eiken Chemical CO., LTD) were followed [10]. The Loopamp kit has been described in detail previously [7]. Briefly:

### Sample Collection and Processing

Fresh blood samples collected by finger prick were immediately used for LAMP. Boil and spin method was used in a sample preparation area separated from the amplification area. An aliquot of 60 μl of whole blood of each patient was transferred to the extraction tube and mixed with 60μl of extraction buffer (400 mM NaCl, 40 mM Tris pH 6.5, 0.4% SDS) by vortex for 10 seconds. Extraction tubes containing the samples were placed in the Hot-block (Eppendorf 5350) at 95°C for 5 minutes, later centrifuged at 10,000 g for 3 minutes (mini-centrifuge MCF-2360) and finally 30μl of clear supernatant were transferred to the dilution tube (if not used immediately, DNA sample should be stored at -20°C).

### LAMP Reaction and Reading

Two primer sets provided with the kit were designed to detect the mitochondrial DNA of Plasmodium parasites. The Pan specific primers detect a target DNA sequence and are able to detect a wide range of Plasmodium species including the four most common ones that cause human malaria. The *P*. *falciparum* (Pf) specific primers have been confirmed to be specific for *P*. *falciparum* parasites.

As it is required to test both for Pan and Pf, two reaction tubes (one for Pan and other for Pf specific primer) per sample were used in each run (10 samples), plus two reaction tubes for control (one reaction tube for positive control (PC) and a different one for negative control (NC)) (both included in the kit) which allowed a total of 24 reaction tubes.

Once DNA was extracted, 30 μl were added in a reaction tube for Pan detection, and 30 extra microliters were added in a different reaction tube for Pf detection. Finally, a NC and a PC were addded in both reaction tubes. All of them were shaken to ensure proper mixing and LAMP reagents dissolving. The tubes were placed immediately into the hot-block at 65°C for 40 minutes (amplification reaction). At the end of amplification reaction, tubes were heated at 80°C for 5 minutes, to terminate the reaction (enzyme inactivation).

The bottom of each reaction tube was irradiated with UV lamp (wave length = 240 nm to 260 nm and 350 nm to 370 nm) and observed from the side through dark glasses. For a valid run, green light is emitted by PC and no light is emitted by the NC.

### Cost and Time-To-Completion Analysis

A cost and time-to-completion analysis for every method used were done according to Shillcutt S et al [11]. Costs of microscopy diagnosis included materials, staff time, training and technical supervision. RDT and LAMP diagnosis included the unit cost of the test; diagnosis according to presumptive treatment was assumed to have no cost.

Costs of an adult ACT (artemether-lumefantrine (Coartem^®^)) dose were worked out from the current whole sale price ($2.40)[12].

We set the cost of RDT kits at US$ 0.6–1 and that of microscopy at US$ 0.32–1.27. Microscopy costs are dependent on workload and were based on a range from 1000 to 6800 or more diagnoses per year. To clarify concepts, we assumed that microscopy was used only for malaria diagnosis, but not for other diseases.

### Statistical Analysis

For the statistical analysis, Software EPIDAT 3.1 was used. Final results of microscopy and RDT were compared to results obtained using LAMP using McNemar’s test; and sensitivity, specificity, positive predictive value (PPV) and negative predictive value (NPV) were calculated with 95% confidence interval (CI). Prevalence results were expressed with a 95% CI.

In the descriptive study, continuous variables were represented as the mean ± standard deviation (SD), and qualitative variables were expressed as relative frequencies. Statistically relation between lamp-positive and a) quantitative diagnostic parameters were made using Mann-Whitney U test and b) qualitative diagnostic parameters using Chi-squared test. A P-value of <0.05 was considered to indicate statistical significance.

## Results

Statistically significant differences among the different methods used comparing with LAMP results were found. Eleven out of the 162 patients with fever tested positive for LAMP (prevalence: 6.8%; 95% CI: 3.8–11.7%), 3 for RDTs (prevalence: 1.8%; 95% CI: 0.6–5.3%) (p = 0.0133 McNemar’s test) and none was detected by thin film microscopy (prevalence: 0%; 95% CI: 0–2.3%) (p = 0.0094 McNemar’s test). Results are shown in Table 1.

**Table 1:** Comparison between LAMP, RDTs and on-site thin film microscopy in LAMP positive patients. *RDT*: *Rapid Diagnostic Test; Mic*: *Microscopy; Hb*: *Haemoglobin (g/dl); Pla*: *Platelets (/μl)*, *m*: *male; f*: *female*, *N negative*, *Pf*: *Plasmodium falciparum*, *Pv Plasmodium vivax*.

Age	Sex	RDT	LAMP	Mic	Hb	Pla
6	m	Pf	Pf	N	8.5	140,000
30	f	N	Pf	N	12.7	25,000
22	f	N	Pf	N	12.8	90,000
22	f	N	Pf	N	5.1	123,000
11	f	N	Pf	N	14.8	153,000
15	m	N	Pv	N	16.5	172,000
10	m	N	Pv	N	11.7	92,000
15	m	Pf	Pf	N	6.6	131,000
38	f	N	Pf	N	7.6	35,000
20	f	N	Pf	N	12.6	379,000
65	m	Pf	Pf	N	12.4	61,000

Sensitivity, specificity, PPV and NPV for RDTs were 27% (CI95%: 0.58%), 100% (95% CI: 99.6–100%), 100% (95% CI: 83.3%) and 94% (95% CI: 91.2–98.6%), respectively.

Table 2 shows a lower level of haemoglobin and platelets count for LAMP positive patients compared with LAMP negative.

**Table 2:** Quantitative diagnostic parameters and qualitative diagnostic parameters for LAMP positive patients.

**Quantitative diagnostic parameters**	**Mean ±SD Positive LAMP**	**Mean ±SD Negative LAMP**	**P-value**
**Age**	23.091±16.670	24.720±14.971	0.593
**Days with symptoms**	5.182±3.573	6.618±3.880	0.186
**Leucocytes**	8054.545±4887.610	8722.302±5031.078	0.397
**Lymphocytes**	2294.027±1789.285	1957.252±1311.938	0.840
**Neutrophils**	5152.400±3267.980	5440.999±4529.712	0.979
**Haemoglobin**	9.900±4.832	13.021±3.226	0.015
**Platelets**	110666.667±43247.351	256869.919±104907.606	≤0.001
**Qualitative diagnostic parameters**	**Positive LAMP Number (%Total Positive)**	**Negative LAMP Number (%Total Negative)**	**P-value**
**Sex(Female)**	6(54.54%)	83(58.041%)	0.821
**Admitted**	2(18.18%)	17(11.18%)	0.667
**Headache**	9(81.82%)	73(48.03%)	0.091
**Nausea**	6(54.54%)	27(17.76%)	0.012
**Shivering**	3(27.27%)	26(17.10%)	0.395
**Other** *	8(72.73%)	81(53.29%)	0.472

*Other symptoms included general malaise, abdominal pain and cough.

The cost-effectiveness of LAMP, RDTs and microscopy relative to presumptive treatment is shown in Table 3.

**Table 3:** A cost and time-to-completion analysis for all the methods used was done.

Method	Cost of diagnosis/sample	Time for diagnosis	treatment cost^3^,^4^	Total cost^5^
**LAMP**	8.18$/sample	1 hour/10 samples^1^	57,6$	1382.60$
**RDT**	0.60–1.00$/sample	20 min-30min/10samples	115.2$	212.4–277.2$
**Microscopy**	0.32–1.27$/sample	30–60 min/sample^2^	158.4$	210.40–364.14$

^1^: DNA extraction takes 10min + LAMP reaction and reading 50min. Capacity of hot-block is 24 samples: each determination comprises two tubes, one for *P*. *falciparum* and one for *Plasmodium spp*.+ controls (positive and negative for *P*. *falciparum* and *P*. *vivax*).

^2^: To obtain the same sensitivity of examination as that for thick film at high power fields (with 100x oil immersion objective) for 10 min, a thin film should be examined for at least 30 min[13].

^3^: Only treatment with ACT for patients with negative diagnoses with each method were considered.

^4^: Treatment with ACT was considered exclusively.

^5^: Total cost of diagnosis + total cost of treatment.

## Discussion

Malaria incidence has been declining globally in the last decade. However, in countries like Ethiopia, it remains as one of the top health problems and has been reported as one of the three leading causes of morbidity and mortality in the past years [3]. Efforts to control and eradicate malaria require a strong diagnostic capability, which should allow a prompt detection and treatment of any parasitemia patient.

Recent studies have shown sensitivity values as low as 51% when performing thick and thin film by expert microscopists in comparison with LAMP assays in Uganda [14]. In a previous study performed in the catchment area of Gambo Hospital in Ethiopia [13], LM (thick and thin film seen analysed by an external observer) had a 52% sensitivity equally, when compared with a semi-nested PCR for the diagnosis of *P*. *falciparum* and *P*. *vivax* malaria. LAMP Kit for malaria diagnosis may be used by laboratory technicians without previous training in molecular methods. Knowledge and skills can be acquired during a short training period of less than three days. However, strict adherence to the procedures is necessary to achieve reliable results. Different clinical studies have validated this rapid molecular test in the field with a performance similar to conventional PCR [15]. Additional advantages of LAMP are its tolerance to inhibitory substances present in blood samples (such as haemoglobin and immunoglobulin) [16] as well as the possibility of being used also on a minimal amount of blood on filter papers [17].

The results obtained in this study indicate the urgent need of improving the quality of light microscopy in non-certified clinical laboratories and providing RDTs to hospital laboratories with substandard proficiency in malaria microscopy, at least until the laboratory achieves a certification. The poor sensitivity showed by RDTs in comparison with other field studies performed across Africa could indicate that in our small group most positive patients were infected with very low parasitemia (< 200 trophozoites/microliter). Results obtained by the WHO in 2013 showed a 5.7%-86.0% of panel detection score for *P*. *vivax*-*P*. *falciparum* and 0% of positive test results for *P*. *falciparum* in Pan line, with this density [18]. Moreover, false-negative RDT results are associated with deletions of HRP-2 and HRP-3 genes[1]. Cook et al showed that LAMP detected a higher number of infection compared with RDT in Zanzibar (18 against 10 infections)[19].

In accordance with the studies of Patel et al in Thailand and India or Aydin-Schmidt in Zanzibar, this study shows that LAMP can be used in a rural and remote area to detect the presence of very low parasitemia patients not diagnosed by RDTs as well as the convenience of using more powerful diagnostics tools in any strategy aimed to control or eradicate malaria in low transmission areas [15, 17, 20–21]. Mainly because of its cost, we agree with the experience of Hsiang et al in Thailand and India: LAMP should probably not be used as a point of care diagnostic tool for symptomatic individuals in most resource-limited settings. Rather, LAMP should generally be reserved for active surveillance of subpatent infections [22]. Another potential use of this technique in the field could be detecting infected blood donors before transfusion.

As previously shown, thick film microscopy and RDTs both become relatively insensitive at parasite densities below 100 parasites/μl. Current surveillance systems which rely on microscopy or RDTs are not sensitive enough to detect asymptomatic and paucisymptomatic carriers who act as reservoirs for malaria transmission and may develop malaria episodes in the future [1, 23]. Furthermore, co-infections of multiple species complicate malaria diagnosis in rural field settings and in very well supplied laboratories [1].

Port el al indicated an important limitation, which is the problem of potential unavailability of electricity used for irraditation and heating of thermo-blocks in the field. However, trials using a hand-held battery operated thermoblock were carried out and offered a cheap, simple and fast method of molecular detection of malaria parasites.[8]. Moreover, the use of LAMP in the dectection of Plasmodium DNA in saliva and urine samples provides a new approach for malaria diagnosis. Ghayour et al showed that a correlation between the parasitemia and the transfer of malaria DNA to saliva and urine exists [9].

LAMP may provide reliable results in basic rural laboratories without the need for specialized infrastructure and can represent a powerful tool in malaria control programs.
